# Epistasis for Growth Rate and Total Metabolic Flux in Yeast

**DOI:** 10.1371/journal.pone.0033132

**Published:** 2012-03-06

**Authors:** Agata Jakubowska, Ryszard Korona

**Affiliations:** Institute of Environmental Sciences, Jagiellonian University, Krakow, Poland; Centre for Genomic Regulation, Spain

## Abstract

Studies of interactions between gene deletions repeatedly show that the effect of epistasis on the growth of yeast cells is roughly null or barely positive. These observations relate generally to the pace of growth, its costs in terms of required metabolites and energy are unknown. We measured the maximum rate at which yeast cultures grow and amounts of glucose they consume per synthesized biomass for strains with none, single, or double gene deletions. Because all strains were maintained under a fermentative mode of growth and thus shared a common pattern of metabolic processes, we used the rate of glucose uptake as a proxy for the total flux of metabolites and energy. In the tested sample, the double deletions showed null or slightly positive epistasis both for the mean growth and mean flux. This concordance is explained by the fact that average efficiency of converting glucose into biomass was nearly constant, that is, it did not change with the strength of growth effect. Individual changes in the efficiency caused by gene deletions did have a genetic basis as they were consistent over several environments and transmitted between single and double deletion strains indicating that the efficiency of growth, although independent of its rate, was appreciably heritable. Together, our results suggest that data on the rate of growth can be used as a proxy for the rate of total metabolism when the goal is to find strong individual interactions or estimate the mean epistatic effect. However, it may be necessary to assay both growth and flux in order to detect smaller individual effects of epistasis.

## Introduction

The study of epistasis has long been divided between detailed examination of functional interactions between selected genes and statistical detection of biases in the inheritance of polygenic traits [1]. Systems biology promises to cancel this division by extending the functional study on all possible interactions and expressing the results in a quantitative manner [2]. The pursuit is currently most advanced in case of the budding yeast. Data are obtained mostly through large scale automated assays and therefore often suffer from a relatively high rate of false negatives or positives [3], [4], [5]. Nevertheless, some of the already available results appear robust. Interactions leading to strong decreases or increases in fitness of double mutants in relation to that of respective single mutants are generally rare and amount to a half or a few percent depending how stringent criteria are adopted [4], [5], [6]. Weak interactions are more abundant with an average effect being close to zero or moderately positive [4], [5], [6], [7], [8], [9]. Therefore epistasis is unlikely to intensify selection against deleterious mutations [10], [11]. Although limited to one species maintained under laboratory conditions, these conclusions provide an important example that aggregated results of large-scale functional studies can indeed provide quantitative answers to some long standing problems of evolutionary biology.

A possible caveat is that the rate of growth is used as a sole measure of fitness in the large-scale studies. Positive or negative effects of gene interaction can be significant in other key components of fitness, for example, the efficiency of resource utilization. It is especially important to have possibly broad measure of fitness when an average epistatic effect is considered because even small biases in its value can change expectations on the evolution of genetic recombination and sexual reproduction [12], [13], [14]. It may appear that the efficiency of growth is of little significance. To grow faster, the cell typically switches from respiration to fermentation reducing radically the number of ATPs obtained from glucose. In microorganisms this means throwing usable byproducts out of the organism, the budding yeast excels in this [15], [16]. In effect, the ratio of dry mass to consumed glucose goes from about 50% under purely respiratory metabolism to only 10% as fermentation takes over [17]. However, glucose is needed not only to generate energy but also to acquire blocks for the synthesis of biomass. It seems that to enhance the rate of growth the efficiency of energy generation must be lowered. This can be a major trade off in cellular economics as a single type of metabolism fitting all environments apparently does not exist [18]. The efficiency of growth is important even when growing in good conditions at maximum speed because natural habitats of yeast are probably often fragmented and populations are likely structured. and therefore it matters how much cells (biomass) is produced from available resources. Indeed, yeast appears especially well adapted to grow in good conditions, it can evolve adaptation to low glucose but pays for it with a lowered performance in high glucose [19].

Cells of the budding yeast growing in the presence of glucose present at sufficiently high concentration metabolize it solely by fermentation to ethanol. Furthermore, glucose prevents any use of the accumulating ethanol and this is assured by allosteric regulation of enzymes and strong control of gene expression [20]. When the concentration of glucose is above 1%, the capacity of the cell to uptake it and secure as phosphorylated hexoses is twice higher than required by the downstream metabolism [21], [22]. Thus, the cells are tuned to a strictly defined type of metabolism and fed ‘ad libitum’. This is a very special and experimentally favorable situation in which simple measurements of the glucose consumption rate are likely to provide estimates of the global metabolism intensity that are straightforwardly comparable between deletions. Individual strains represent effects of specific metabolic distortions within the same general physiological makeup. The glucose-dependent regulation is so basic for the budding yeast that we assume it operates similarly in all mutants used here as long as glucose is abundant and growth exponential. For these reasons we confined our experiments to conditions promoting the fermentative metabolism.

We asked how single or double gene deletions change not only the tempo of growth but also the intensity of metabolism needed to accomplish it. Technically, we measured the maximum rate of growth and the rate of total metabolism (flux) approximated as glucose consumed per synthesized biomass. We found that the two rates were related linearly and, as a result, the mean epistatic effects on growth and flux tended to mirror each other.

## Results

### Different gene deletions have different effects on the metabolic efficiency

In the present experiments, virtually all biomass was produced in the phase of exponential growth. Thus, biomass *B* was produced from glucose *G* with a constant efficiency of conversion *c*, so that *B = cG*. An equation describing growth of a biomass with an intrinsic rate *r*, *dB/dt = rB*, can be rewritten as *dB/dt = cgB*. In this way, *g* stands for the rate of glucose uptake in relation to the growing biomass (as a proxy of the intensity of metabolism). We measured empirically *r* and *c* of individual strains and then approximated their *g* as *r*/c.

**Figure 1:**
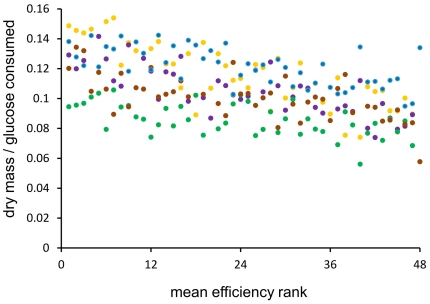
Metabolic efficiency in different environments. A sample of single deletion strains was assayed for dry mass and glucose consumption. The strains were ranked within a single environment and then a mean rank was calculated. Data points are shown for: YPD (blue), 37°C (yellow), caffeine (violet), sodium chloride (brown), and minimal SD medium (green).

We first asked whether strains differ in *c*, the coefficient of efficiency, and whether the differences are substantially high and independent of specific environmental conditions. A sample of 48 yeast strains with single deletions of functionally different genes were grown in batch cultures in 5 different media. The choice of deletions for this and the next experiment was generally random but it turned out that the samples' Gene Ontology Slim terms fit well the whole genome profile in terms of biological process, function, and cellular localization (see Materials and methods and Tables S1 and S2). The concentration of glucose was initially at 2%, the cells were harvested before it came down to 1%. As expected, these conditions yielded dry mass equal roughly to one tenth of consumed glucose. This is shown in Fig. 1 together with the evidence that this efficiency of glucose conversion varied considerably between different environments (F = 59.879, df = 4, P«0.001). This was not surprising as more resources are needed under high concentration of salt which stimulates a costly reaction to the osmotic stress or in minimal medium in which anabolism is more intense than in rich medium. There were also significant differences in the efficiency between individual deletion strains (F = 4.216, df = 40, P«0.001; strains with lethal phenotypes in any environment were excluded from the ANOVA analysis). The differences were remarkably consistent: strains tended to have either low or high efficiencies in all five tested environments. In every environment, the differences in efficiency reached some 10–15% of an average value (Fig. 1). In sum, single gene deletions could lead to a noticeable although not overwhelming decrease in the metabolic efficiency of the yeast cell. Furthermore, relative efficiencies of different strains were generally stable over different environments.

### Patterns of epistasis for growth and flux are similar

In this experiment we used strains derived from crosses between two single deletion strains of different markers, geneticin or nourseothricin. A single mating followed by meiosis and sporulation yielded a strain without deletions, two strains with single deletions and a strain with two deletions. Estimates of *r* and *g* were obtained for 384 strains descending from 96 crosses between pairs of unique gene deletions. Assays were done in single environment, YPD and 30°C. We asked how the rate of growth and the metabolic flux change with the number of gene deletions. We specifically looked for epistasis between two gene deletions in relation to the rate of growth, *r*, and the rate of glucose uptake, *g*. The effect of epistasis for the growth rate was calculated as *ε_r_* = (*r_0_*+*r_kn_*)−(*r_k_*+*r_n_*) where *k* and *n* stand for deletions marked with genes coding for the geneticin and nourseothricin resistance respectively; *ε_g_* was calculated analogously. We use a normalized version of the above equation (after dividing all rates by *r_0_*) while reporting our results. Null values of *ε* indicate an absence of epistasis, that is, additivity of log-fitness (i.e. the rate of reproduction) which is equivalent to multiplicity of fitness (the realized reproduction).

**Figure 2:**
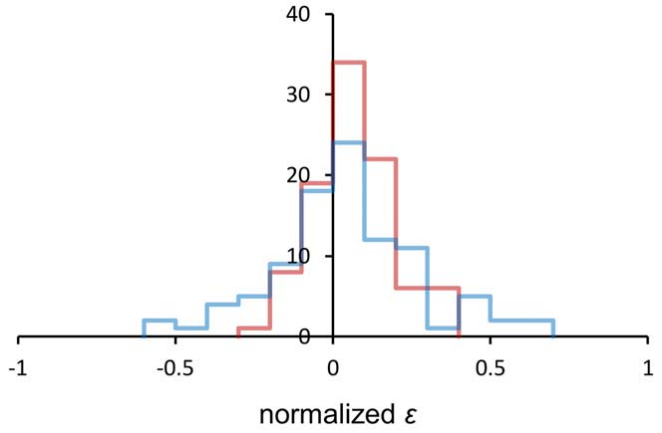
Frequency distribution of the epistatic effect. Epistasis for the rate of growth (*ε_r_*) and the flux of glucose (*ε_g_*) are shown in red and blue, respectively. Normalization was done by setting the mean growth rate (*r*) or flux (*g*) of the strains with no deletions to zero.

Fig. 2 presents the frequency distribution for *ε_r_* and *ε_g_*. The two means were 0.0623 and 0.0441, respectively. They were not different from each other (*t* = 0.699, df = 190, *P* = 0.504); *ε_r_* was significantly higher than zero (*t* = 4.855, df = 95, *P*«0.001), *ε_g_* was not (*t* = 1.828, df = 95, *P* = 0.071). Note that not only the means but also shapes of the two distributions were similar except that the distribution of *ε_g_* was clearly wider. This is understandable because to estimate *g* additional errors arose as both glucose uptake and dry mass buildup had to be estimated. We suggest that high dispersion, not low mean value, could be responsible for the failure to show that also the epistasis for flux was positive.

Some researchers estimate epistasis from the expression *r_0_r_kn_*−*r_k_r_n_* even though log-fitness (the rate of growth) is normally combined additively [23]. We nevertheless used this “super-multiplicative” model to re-calculate epistasis for the rate of growth and flux and found their mean values at 0.0268 and 0.0211, respectively. The two means were not different in statistical terms (*t* = 0.244, df = 190, *P* = 0.807); epistasis for the rate of growth was significantly higher than zero (*t* = 2.596, df = 95, *P* = 0.011) while that for flux was not (*t* = 1.008, df = 95, *P* = 0.316). Thus, both models led to identical conclusions of which the most important is that an overall effect of interaction between two gene deletions appeared similar when two important phenotypic traits, the rate of growth and the rate of total metabolic flux, were compared.

### Metabolic efficiency is largely independent of the rate of growth

**Figure 3:**
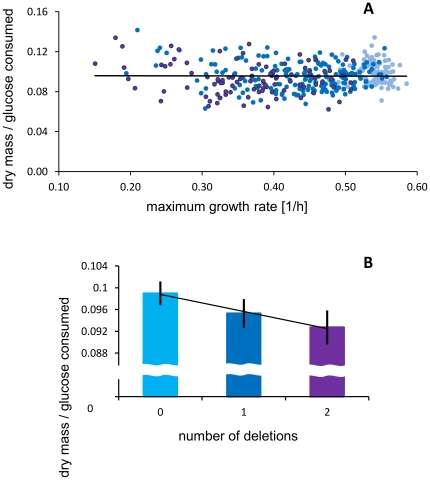
Metabolic efficiency. (A) The maximum growth rate (*r*) and the efficiencies (*f*) of strains with none (light blue), single (blue), and double gene deletions (violet) are shown. The overall regression line is *f* = 0.0962-0.0014*r*, and is statistically indistinguishable from being flat (*t* = 0.184, df = 382, *P* = 0.855). (B) The mean efficiencies with 95% confidence limits for the three groups respectively.

One possible explanation why the epistasis for flux matched that for growth was that an average coefficient of efficiency *c* did not change or changed very little with the rate of growth. If so, *c* would be basically a constant scaling factor, *g* = *r/c*. Indeed, Fig. 3a shows a striking stability of *c* over a wide range of the growth rate. A possible caveat could be that the efficiency was estimated so imprecisely that this obscured any existing trend. But, each point presented in the figure was based on two independent estimates (see Methods). These tended to correlate with each other so that the squared Pearson's coefficient was 0.359 (F = 213.746; dfs = 1, 382; P<<0.001) indicating that repeatability of the estimates explained a sizable portion of variation.

**Figure 4:**
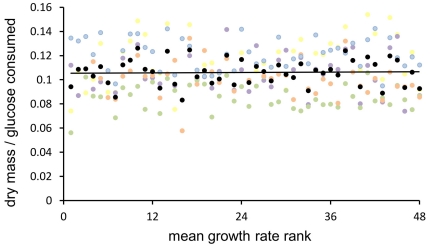
Metabolic efficiency and the rate of growth in five environments. The maximum growth rate was measured for the sample of 48 strains and used to rank strains within each environment. Black points represent growth ranks averaged over 5 environments, with a regression line with a slope of 0.00005 which is statistically indistinguishable from being flat (*t* = 0.221, df = 46, *P* = 0.826).

Considering the number of deletions, it appears that the more deletions the lower *c* although the decreases are small, no larger than a few percent (Fig. 3b). To further investigate the relation between the rate of growth and its efficiency, we returned to the strains formerly tested in five different environments. After measuring their growth rate in every environment, we ordered them according to their average growth rate rank (Fig. 4). This new arrangement confirmed that the efficiency of growth was independent of its rate, this conclusion held for all tested environments.

The above results appear somewhat contradictory. On one hand the efficiency of resource conversion showed strong signs of stability in relation to the rate of growth, on the other there were some differences between strains with 0, 1, or 2 deletions. The differences were small, might be spurious or caused by the additions of markers, not deletions of genes. Note, that even if real they were proportional to the number of deletions (Fig. 3b). It means that the mean value of *c* and *c_kn_* and that of *c_k_* and *c_n_* were close to each other and therefore tended to cancel out from the formula for *ε*. Thus, even if *c* was not entirely independent of the number of deletions it did not change our main conclusion that that the average effect of epistasis for *g* must be close to that for *r*.

### Individual effects of gene deletions on metabolic efficiency are heritable

**Figure 5:**
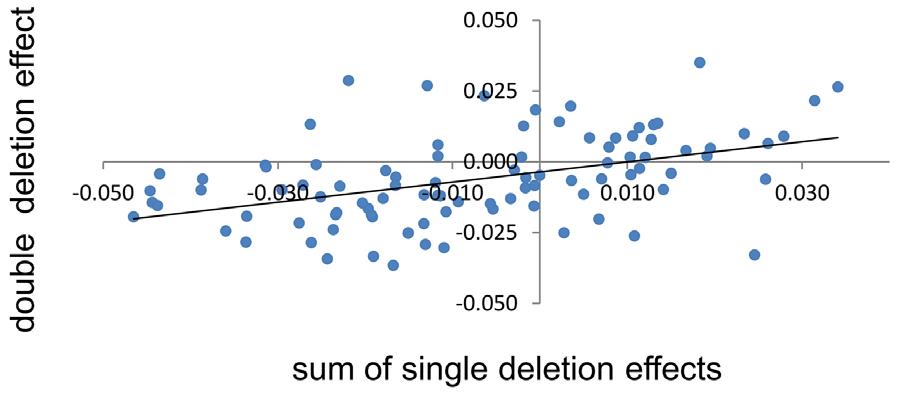
Genetic transmissibility of the metabolic efficiency. The horizontal axis represents sums of the efficiency effects of a pair of strains with single deletions (*s*), the vertical one represents the efficiency effects of strains with the two respective deletions (*d*). The efficiency effect was calculated as a distance from the mean efficiency of 96 strains with no deletions. The linear regression is: *d* = 0.357*s*–0.00362. The slope of the regression line is statistically different from zero: *t* = 4.867, *df* = 94, *P*«0.001.

Our results reveal a special pattern of pleiotropy. A single deletion can affect both the rate of growth and the efficiency of converting glucose into biomass but the two traits do not correlate with each other. Being unrelated to such an important trait as the rate of growth, the efficiency of glucose conversion could be an unstable or, in genetic terms, poorly heritable phenotype. To test it, we first calculated differences between individual *c*'s and the average *c* of the 96 strains without deletions (efficiency effects). We then regressed the efficiency effects of double deletions over the sums of efficiency effects of relevant single deletions (Fig. 5). Estimated as the slope of the regression line, heritability was equal to 0.357. In conclusion, the efficiency of glucose conversion is a transmissible trait and thus can be controlled by natural selection.

## Discussion

Some metabolic parameters are likely to be important fitness components under a broad array of external conditions. The ability to grow at possibly highest rate is undoubtedly one of them and was extensively used as a proxy for fitness. In this work we concentrated on the efficiency of converting resources into biomass and showed that it can be markedly different for different yeast gene deletions. A crucial finding is that the efficiency of resource utilization is unrelated to the rate of growth. Therefore, the total flux of metabolites and energy is on average linearly related to the rate of growth. As a result, the average growth effects of both single and double gene deletions are reflected by the average effects on flux, and so is the average epistasis.

The average epistasis for the rate of growth was found moderately positive not only here but also in former studies when a sample of gene deletions was considerably larger. These experiments were based on manual crosses and tetrad analyses leading to complete and balanced sets of strains with zero, one, and two deletions; assays of the maximum growth were done in individual liquid cultures [7]. Other and substantially larger studies applying automated strain construction followed by culturing on agar surfaces suggested that the distribution of epistatic effects has a modal peak close to zero. The latter design is correct when the goal is to find strong effects of gene interaction but possibly less so when small biases in the central tendency are to be determined. This is because automated crosses produce the double deletions only. The no-deletion and single-deletion strains are saved from possible effects of mutation and selection operating in this process. Furthermore, growth on agar surfaces is influenced by neighboring colonies therefore the final size of a colony can depend not only on the rate of growth but also on metabolic efficiency although relative strength of these two factors is unknown. For this reason, data have to be extensively corrected for the plate and position effects [4], [5]. But, despite the described methodological differences none of the previous studies suggested that negative interactions for fitness are common or strong enough to push the mean epistatic effect below zero. The present study upholds and extends this conclusion by showing that the strength and direction of average epistasis for the growth rate is not significantly changed when the growth efficiency is taken into account. This insight can be relevant not only for yeast because signals of growth at the cellular level are similar among eukaryotes [24], [25]. The rate of biomass growth, however, is only one aspect of fitness, especially in more complex organisms. Indeed, epistasis for fitness in multicellular eukaryotes has been found variable, its mean value can be either negative, positive, or close to zero [14], [26], [27], [28].

Our data show that the efficiency of resource utilization is a heritable phenotypic trait of potentially significant impact on fitness and thus worth further study. At the same time, the efficiency of growth is not likely to replace the rate of growth as a trait of choice in the genome-wide studies of single or multiple mutation effects. Not only because its measurements consume more work and are more prone to error but also because the trait is less sensitive to genetic damage. The rate of growth can be more than halved by mutations while the efficiency is usually affected by about one tenth. When the growth effects are strong the flux effects will follow them making their measurements largely unnecessary. On the other hand, small or even none changes in the rate of growth can be associated with sizable changes in its efficiency and therefore the latter may provide valuable information about functioning of genetic networks. Our sample was too small for any comprehensive analysis of factors influencing the efficiency of resource utilization. Unfortunately, massive assays of the growth efficiency will not be easy. The best way of doing them is to harvest cells in truly exponential phase of growth in amounts large enough to measure reliably their dry mass and simultaneously assay how much glucose was used up. Measurements of optical density after cessation of growth can be easily done for the whole gene deletion collection [29]. Estimates obtained in this way sum the amounts of biomass built both before and after the diauxic shift (a phase of switch from fermentation to respiration) whereas relative contributions of the two types of metabolism are usually unknown. Neither the concentration of metabolic products (ethanol, acids) remaining in medium is known. Moreover, the same yeast biomass can have markedly different optical density when the average cell size differs (see Methods and Figure S1). It is well known that size differences are common among deletion strains [30]. Acknowledging these uncertainties, we note that the data based on OD readings of the stationary state which were completed for all gene deletions do not suggest that there might be correlation between the growth rate and efficiency and thus are in line with our results obtained with a smaller sample [31].

It is intriguing why the efficiency of metabolism does not correlate with the rate of growth. Some recent studies suggest that investments in the machinery needed for growth are undertaken not when the cell is really capable of growing fast but when the signals perceived from external environment suggest so [32], [33]. It is thus possible that even though the strains were genetically different their basic makeup and functioning was similar because nutritional signals were homogeneous. Unfortunately, reliable experimental estimates of the costs of growth in relation to other activities of the cell are scarce even for “wild-type” strains [34], [35], [36]. The present study does not answer these basic questions but nevertheless offer some reassuring conclusions. The currently accessible flux-balance models of metabolic networks have been used to investigate a wide array of problems including the functional explanation of gene interaction [5], [37], [38]. These efforts were often successful even though the models were criticized as abstract because of detaching the fluxes from the costs of functioning of the whole cellular hardware [18]. Our results suggest that this can be actually an allowable practice even though it remains unsure why damages that are apparently serious to the rate of metabolism be mild or non-existent to its efficiency.

## Materials and Methods

### Strains

We used some of strains developed in a former study [7]. Briefly, that study started with haploid yeast deletion collections of two opposite mating types (BY4741 and BY4742). In a half of the studied sample, the original marker of resistance to geneticin was exchanged for resistance to nourseothricin. This allowed for mating and tetrad analyses in which a single pair of geneticin and nourseothricin resistant parents yielded four progeny strains: with no marker, with one of single markers, and with double marker. From about seven hundred crosses done in this way, 96 were chosen for the present study. The choice was random, the only exception was that ORFs with unsure protein products were removed. The list of 192 parental strains was then confronted with the list of 4,990 genes with verified protein products. Both lists were compared according to the categories of the Gene Ontology Yeast Slim classification of biological process, molecular function, and cellular location. Pearson's correlation coefficients between frequencies calculated for the sample and the genome were 0.935, 0.942, and 0.964 for the three mentioned classifications respectively. The 192 gene deletions used in the crosses are listed in Table S2. Of these, a sample of 48 were used in the experiment in which the metabolic efficiency was measured in 5 different environments. They are listed in Table S1.

### Growth rate, glucose concentration, and dry mass

The sample of 48 strains were cultured in four different media maintained at 30°C: YPD (broth medium), SD (defined medium), YPD with 7 mM caffeine, and YPD with 0.8 M sodium chloride. The fifth environment was YPD at 37°C. Aliquots of 20 ml were inoculated with 0.25% of an overnight culture and incubated with agitation. Growth was monitored by taking 0.2 ml sample every 40 min. and measuring OD. The maximum growth rate, *r* thorough this paper, was estimated using measurements falling between 3 and 30% of an overnight culture after log-normal transformation. This yielded no less than six time-points and an excellent linear fit. To assay glucose uptaken *G* and dry biomass produced *B*, we collected samples of 6 ml of cultures growing exponentially and reaching 25 to 35% of a typical overnight density. The samples were rapidly cooled down and frozen. Thawed samples were centrifuged, pellets were washed three times, vacuum dried, left at 60°C for further drying over five days, and finally weighed. Supernatants were diluted and subject to assays of glucose with an enzymatic kit D-Glucose-HK (Megazyme) and Tecan Infinite 200 plate reader of fluorescence. Some strains did not grew in certain environments. They were excluded from analyses because assigning null values to both the rate of growth and the efficiency of growth would produce spurious correlation between the two traits.

The sample of 384 strains (96 with no deletions, 192 with single deletions, and 96 with double deletions) was treated in somewhat different way. Growth in YPD at 30°C was monitored in an automated microbiological station Bioscreen C which incubates, agitates, and periodically measures OD of 0.3 ml microcultures. The maximum growth rate was calculated for a similar range of densities as that for the 48 strains sample. The sample of 384 strains were also used to obtain estimates of glucose consumption and biomass buildup. Larger cultures of 5 ml volume were used for these assay. A single sample was taken when the cultures reached 25 to 35% of an overnight density, OD was measured, cells removed, and the supernatant used to assay glucose. The OD reading were used to estimate dry mass, *B*. The relation between OD and dry mass was determined for a representative sample of deletion strains (Figure S1).

All assays were duplicated. Data points in Figures 1, 2, 3, 4 and 5 represent means of two independent estimates.

## Supporting Information

Figure S1 **Estimating dry mass (DM) from optical density (OD).** A sample of 13 deletion strains was brought to exponential growth in the YPD medium. Cultures were rapidly cooled down and re-suspended to form a gradient of 6 densities per strain. DM was related to the maximum growth rate (MGR) and OD of cultures with the least square method (Statistica 9) yielding the following formula: *DM = 0.3126−1.5264×MGR+1.4294×OD+1.9496×MGR^2^−1.0118×MGR×OD+0.6440×OD^2^*. Conclusion: OD underestimates DM when cells are small due to low MGR.
(PDF)

Table S1 **Efficiency (dry mass/glucose) and maximum growth rate of selected strains.** The table lists strains tested in five environments. Empty fields mark conditional lethality in a particular environment.
(PDF)

Table S2 **Efficiency (dry mass/glucose) and maximum growth rate of all strains used in the study.** The table lists strains harboring one deletion (kan or nat), two deletions (kan nat), and no deletions (wt) resulting from 96 crosses.
(PDF)
