# Supplementary figures and images for: Epistasis for Growth Rate and Total Metabolic Flux in Yeast
Source: PLoS One. 2012 Mar 6;7(3):e33132. doi: 10.1371/journal.pone.0033132 (PMC3295780; doi:10.1371/journal.pone.0033132)

Sup. Fig 1

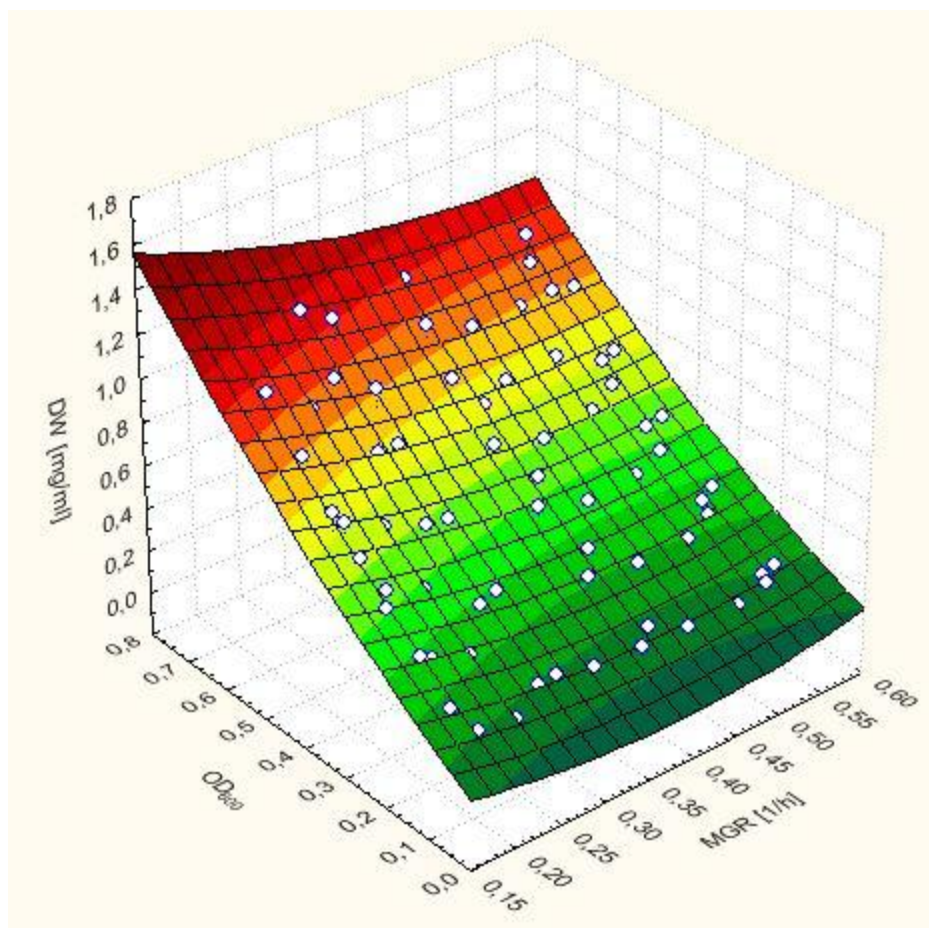

Supplement: Figure S1 — Estimating dry mass (DM) from optical density (OD). A sample of 13 deletion strains was brought to exponential growth in the YPD medium. Cultures were rapidly cooled down and re-suspended to form a gradient of 6 densities per strain. DM was related to the maximum growth rate (MGR) and OD of cultures with the least square method (Statistica 9) yielding the following formula: DM = 0.3126−1.5264×MGR+1.4294×OD+1.9496×MGR2−1.0118×MGR×OD+0.6440×OD2. Conclusion: OD underestimates DM when cells are small due to low MGR. (PDF) [file pone.0033132.s001.pdf]
