# Supplementary material for: Epistasis for Growth Rate and Total Metabolic Flux in Yeast
Source: PLoS One. 2012 Mar 6;7(3):e33132. doi: 10.1371/journal.pone.0033132 (PMC3295780; doi:10.1371/journal.pone.0033132)
Supplement: Table S1 — Efficiency (dry mass/glucose) and maximum growth rate of selected strains. The table lists strains tested in five environments. Empty fields mark conditional lethality in a particular environment. (PDF) [file pone.0033132.s002.pdf]

	Dry mass / Glucose consumed					Maximum Growth Rate [1/h]				
Deletion	YPD	YPD 36C	SD	Saline	Caffeine	YPD	YPD 36C	SD	Saline	Caffeine
YDR347W	0.1132	0.0918	0.0777	0.0795	0.0924	0.4041	0.4199	0.2721	0.2890	0.2624
YMR287C	0.1111	0.1079	0.0768	0.0801	0.0948	0.4431	0.5198	0.3378	0.1835	0.2845
YPR101W	0.1381	0.1487	0.0945	0.1292	0.1202	0.3123	0.3268	0.3186	0.1967	0.2069
YEL024W	0.1331	0.1540	0.1058	0.1119	0.0895	0.4235	0.4409	0.3145	0.2663	0.3090
YKR024C	0.1340		0.0577		0.0577	0.4118		0.3192		0.2347
YEL050C	0.1031	0.1139	0.0691	0.0930	0.1067	0.3715	0.3893	0.2291	0.2139	0.2535
YCR081W	0.1267	0.1375	0.0755	0.1006	0.1029	0.2507	0.5421	0.2548	0.1967	0.1430
YEL054C	0.1278	0.1456	0.0956	0.1199	0.1344	0.3353	0.2904	0.3243	0.2012	0.2152
YAL026C	0.1111	0.0916	0.0911	0.1245	0.1037	0.3298	0.3367	0.3449	0.3010	0.2252
YNL138W	0.1212	0.1175	0.1036	0.1416	0.1176	0.4182	0.3070	0.3073	0.1945	0.2165
YPL234C	0.1156	0.1135	0.1025	0.0995	0.1031	0.3996	0.4537	0.3397	0.1806	0.1513
YHL027W	0.1208	0.1004	0.0864	0.0922	0.1150	0.2872	0.3634	0.2456	0.1621	0.1698
YJR134C	0.1321	0.1069		0.0869		0.2982	0.2108		0.0914	
YER014C-A	0.1188	0.1228	0.0752	0.1190	0.0954	0.4281	0.4691	0.3421	0.2758	0.3443
YHR026W	0.1245	0.1300	0.0798	0.1119	0.0967	0.3230	0.4509	0.3540	0.3569	0.2312
YFR001W	0.1303	0.1304	0.0862	0.1269	0.1063	0.3888	0.4314	0.2984	0.2755	0.3105
YKR092C	0.1262	0.1269	0.0773	0.1086	0.0806	0.3851	0.3448	0.4181	0.2701	0.2838
YAL021C	0.1422	0.1465	0.1010		0.1049	0.3519	0.4075	0.2596		0.2833
YDR495c	0.1233	0.1072	0.0981	0.1016	0.1034	0.3170	0.3475	0.2175	0.2738	0.2163
YLR448W	0.1390	0.1301	0.0858	0.0982	0.1012	0.3090	0.2755	0.2737	0.0863	0.2426
YMR060C	0.1104	0.1102	0.0796	0.0941	0.1010	0.4253	0.4266	0.2503	0.1877	0.2689
YBR251W	0.1028	0.1122	0.0964	0.1024	0.1242	0.3538	0.4238	0.2344	0.2152	0.2350
YDL191W	0.1353	0.1231	0.0817	0.1163	0.1046	0.3758	0.3826	0.2894	0.2718	0.2579
YDL192W	0.1173	0.1238	0.0761	0.1084	0.0836	0.3951	0.4408	0.2987	0.2292	0.2313
YDR028C	0.0949	0.1003	0.0850	0.0815	0.0829	0.4045	0.4945	0.3177	0.2961	0.2796
YMR138W	0.1379	0.1320	0.0877		0.1073	0.3793	0.2115	0.3494		0.0697
YHL033C	0.1064	0.1053	0.0720	0.0967	0.0849	0.3174	0.3839	0.2885	0.1811	0.2394
YNL025C	0.1073	0.1073	0.0823	0.0913	0.0905	0.3607	0.3123	0.3424	0.1793	0.2433
YLR373C	0.1226	0.1208	0.0794	0.1045	0.1005	0.4128	0.4309	0.3267	0.2152	0.2483
YBR126C	0.1183	0.1374	0.0953	0.1359	0.0945	0.4653	0.4538	0.4242	0.2308	0.3117
YAL009W	0.1348	0.1515	0.0794	0.1265	0.1063	0.4393	0.5330	0.3701	0.2077	0.2917
YMR224C	0.1371	0.1219	0.0836	0.1087	0.0886	0.3927	0.3628	0.4106	0.2440	0.2536
YBR127C	0.1080	0.1073	0.0995	0.1011	0.0963	0.3622	0.4081	0.3129	0.1584	0.1868
YPL178W	0.1231	0.0976	0.0779	0.0903	0.0996	0.4836	0.1987	0.3258	0.1945	0.2213
YGL168W	0.0966	0.0896	0.0685	0.0892	0.0838	0.3607	0.2583	0.2730	0.2539	0.1555
YBR073w	0.1193	0.1334	0.0742	0.1184	0.1208	0.3675	0.4149	0.3084	0.2590	0.2698
YLR068W	0.1241	0.1232	0.0934	0.1179	0.1029	0.3058	0.3046	0.2702	0.2071	0.2551
YIL009C-A	0.1418	0.1223	0.0943	0.1083	0.1169	0.3710	0.3021	0.2878	0.2386	0.2783
YLR087C	0.1424	0.1382	0.0825	0.0995	0.1011	0.4275	0.4257	0.3063	0.2805	0.3433
YEL051W	0.1075	0.0897	0.1075		0.0853	0.3305	0.4019	0.3129		0.1819
YER068W	0.1345	0.0741	0.0561	0.1120		0.2304	0.1444	0.2006	0.1101	
YHR183W	0.1221	0.1439	0.0968	0.1255	0.1320	0.3841	0.4781	0.3227	0.2601	0.2629
YGL219C	0.1132	0.1071	0.0947	0.1282	0.1118	0.4216	0.4244	0.2970	0.1905	0.2331
YBR289W	0.1358	0.0891	0.1022			0.1324	0.3351	0.1718		
YLR039C	0.1040	0.0755	0.0911	0.0951	0.1161	0.4514	0.1776	0.2867	0.1871	0.2015
YCR071C	0.1122	0.0918	0.0866	0.0870	0.0856	0.4774	0.5025	0.3248	0.3406	0.2861
YML032C	0.1056	0.1065	0.0864	0.1070	0.0977	0.3577	0.4310	0.2654	0.2698	0.1512
YMR035W	0.1115	0.1047	0.0834	0.0739	0.0942	0.4396	0.5423	0.3269	0.2729	0.3017
