# Supplementary material for: Epistasis for Growth Rate and Total Metabolic Flux in Yeast
Source: PLoS One. 2012 Mar 6;7(3):e33132. doi: 10.1371/journal.pone.0033132 (PMC3295780; doi:10.1371/journal.pone.0033132)
Supplement: Table S2 — Efficiency (dry mass/glucose) and maximum growth rate of all strains used in the study. The table lists strains harboring one deletion (kan or nat), two deletions (kan nat), and no deletions (wt) resulting from 96 crosses. (PDF) [file pone.0033132.s003.pdf]

Cross	Marker	Deletion	MGR	D/G
1	kan	YEL054C	0,4170	0,0972
	nat	YAL026C	0,4587	0,0891
	kan nat		0,3996	0,1009
	wt		0,5631	0,0995
2	kan	YPL050C	0,4868	0,0974
	nat	YML024W	0,5333	0,0998
	kan nat		0,3852	0,0833
	wt		0,5564	0,1008
3	kan	YLR386W	0,5468	0,0971
	nat	YAL021C	0,4180	0,0955
	kan nat		0,5078	0,0822
	wt		0,5426	0,0990
4	kan	YNR036C	0,2377	0,1209
	nat	YMR150C	0,4626	0,0953
	kan nat		0,1790	0,1340
	wt		0,5556	0,0986
5	kan	YAL009W	0,4586	0,1029
	nat	YMR224C	0,4488	0,0856
	kan nat		0,3899	0,0848
	wt		0,5516	0,0992
6	kan	YAL020C	0,5366	0,1024
	nat	YMR228W	0,4976	0,0824
	kan nat		0,4916	0,0697
	wt		0,5514	0,0998
7	kan	YIL103W	0,5005	0,0925
	nat	YMR021c	0,5080	0,0889
	kan nat		0,4577	0,0935
	wt		0,5537	0,0892
8	kan	YPL031C	0,3327	0,1015
	nat	YMR035W	0,4443	0,0930
	kan nat		0,3795	0,0859
	wt		0,4956	0,1173
9	kan	YOL095C	0,4992	0,0885
	nat	YMR287C	0,4522	0,0929
	kan nat		0,3222	0,0906
	wt		0,5303	0,1052
10	kan	YOR026W	0,3856	0,0888
	nat	YNL297C	0,5138	0,0977
	kan nat		0,3883	0,0869
	wt		0,5220	0,1031
11	kan	YPR101W	0,3856	0,1154
	nat	YOL023W	0,4721	0,0820
	kan nat		0,3674	0,0905

	wt		0,5496	0,0962
12	kan	YOR295W	0,3508	0,1138
	nat	YEL024W	0,3128	0,1075
	kan nat		0,2787	0,1089
	wt		0,5505	0,0890
13	kan	YKR024C	0,4018	0,1056
	nat	YEL050C	0,3524	0,0867
	kan nat		0,2792	0,0841
	wt		0,5422	0,0984
14	kan	YDR300C	0,4973	0,0888
	nat	YHR203C	0,4999	0,0974
	kan nat		0,4586	0,0870
	wt		0,5438	0,0956
15	kan	YCR081W	0,4795	0,0913
	nat	YCR003W	0,4361	0,0869
	kan nat		0,3723	0,0824
	wt		0,5470	0,0980
16	kan	YIL065C	0,3203	0,0999
	nat	YLR201C	0,5366	0,0851
	kan nat		0,2567	0,1258
	wt		0,5657	0,1072
17	kan	YMR038C	0,5057	0,0974
	nat	YCR009C	0,5125	0,1076
	kan nat		0,4804	0,0929
	wt		0,5395	0,1030
18	kan	YBR194W	0,3555	0,1143
	nat	YKL113C	0,4877	0,0836
	kan nat		0,3425	0,0941
	wt		0,5434	0,1165
19	kan	YHR021C	0,2355	0,1062
	nat	YPL271W	0,4215	0,1024
	kan nat		0,1506	0,1080
	wt		0,5268	0,1200
20	kan	YNL138W	0,4438	0,1000
	nat	YPL234C	0,4047	0,1014
	kan nat		0,3571	0,1186
	wt		0,5348	0,1097
21	kan	YDL081C	0,5111	0,1037
	nat	YPL174C	0,5257	0,1072
	kan nat		0,5006	0,1120
	wt		0,5341	0,1127
22	kan	YDR432W	0,4681	0,0813
	nat	YBR181C	0,4029	0,0778
	kan nat		0,3286	0,0889
	wt		0,5262	0,0851
23	kan	YPL059W	0,4580	0,0895
	nat	YDR069C	0,4327	0,0974
	kan nat		0,3211	0,0686
	wt		0,4961	0,1017

24	kan	YOL076W	0,4031	0,1188
	nat	YDR120C	0,5129	0,0895
	kan nat		0,3373	0,0943
	wt		0,5280	0,0940
25	kan	YPL129W	0,4298	0,0774
	nat	YDR127W	0,4476	0,0819
	kan nat		0,3532	0,0928
	wt		0,5366	0,1260
26	kan	YJL176C	0,4854	0,0878
	nat	YDR377W	0,4886	0,0830
	kan nat		0,3852	0,0907
	wt		0,5337	0,0890
27	kan	YHL027W	0,3181	0,1073
	nat	YDR378C	0,3988	0,0983
	kan nat		0,2853	0,0986
	wt		0,5238	0,1009
28	kan	YOR293W	0,3692	0,0945
	nat	YKL134C	0,4671	0,0797
	kan nat		0,3052	0,0749
	wt		0,5292	0,0886
29	kan	YJR134C	0,4362	0,0828
	nat	YOR205C	0,4805	0,0922
	kan nat		0,4231	0,0903
	wt		0,5505	0,0908
30	kan	YNL284C	0,4900	0,1104
	nat	YLR372W	0,4980	0,1017
	kan nat		0,5072	0,0891
	wt		0,5540	0,1164
31	kan	YAL010C	0,2504	0,1188
	nat	YDR197W	0,4771	0,1050
	kan nat		0,1969	0,0927
	wt		0,5383	0,0990
32	kan	YEL036C	0,5296	0,1093
	nat	YDR231C	0,4928	0,1000
	kan nat		0,4972	0,0966
	wt		0,5376	0,1043
33	kan	YLR234W	0,4362	0,1098
	nat	YPR191W	0,4956	0,1002
	kan nat		0,3995	0,1005
	wt		0,5197	0,0965
34	kan	YER014C-A	0,4721	0,0985
	nat	YHR026W	0,3787	0,1162
	kan nat		0,3694	0,1029
	wt		0,5446	0,1050
35	kan	YFR001W	0,4192	0,1011
	nat	YKR092C	0,4455	0,0951
	kan nat		0,3486	0,0897
	wt		0,4932	0,0963
36	kan	YDR495c	0,2684	0,1215

	nat	YGL084C	0,4624	0,1079
	kan nat		0,3247	0,1205
	wt		0,5299	0,1161
37	kan	YPR159W	0,5022	0,1146
	nat	YJL056C	0,4742	0,0947
	kan nat		0,5109	0,1110
	wt		0,5322	0,1066
38	kan	YGL038C	0,4964	0,1071
	nat	YLR425W	0,4383	0,1100
	kan nat		0,4780	0,1010
	wt		0,5418	0,1076
39	kan	YDR225w	0,4297	0,0867
	nat	YLR448W	0,2410	0,1240
	kan nat		0,2431	0,1068
	wt		0,4923	0,1021
40	kan	YMR214W	0,5206	0,0941
	nat	YMR060C	0,3448	0,0974
	kan nat		0,2674	0,1222
	wt		0,4991	0,0975
41	kan	YOR270C	0,4695	0,0851
	nat	YJR104C	0,4807	0,0865
	kan nat		0,4587	0,1122
	wt		0,5601	0,1047
42	kan	YBR251W	0,3612	0,1112
	nat	YDL191W	0,4198	0,1129
	kan nat		0,2483	0,1054
	wt		0,5273	0,1059
43	kan	YLR423C	0,5545	0,1235
	nat	YDL192W	0,4177	0,1024
	kan nat		0,4154	0,1079
	wt		0,5470	0,0913
44	kan	YDR268W	0,4688	0,1087
	nat	YDR028C	0,4059	0,0876
	kan nat		0,4032	0,0933
	wt		0,5366	0,1065
45	kan	YMR138W	0,4501	0,1014
	nat	YCR024C	0,5001	0,0992
	kan nat		0,4378	0,0738
	wt		0,5617	0,0878
46	kan	YDR138W	0,5283	0,0900
	nat	YCR034W	0,4720	0,1074
	kan nat		0,4363	0,1172
	wt		0,5605	0,1117
47	kan	YHL033C	0,3748	0,1031
	nat	YNL025C	0,4223	0,0919
	kan nat		0,3971	0,0960
	wt		0,5342	0,1125
48	kan	YBR266C	0,5200	0,0996
	nat	YNL037C	0,4943	0,0965

	kan nat		0,4485	0,1116
	wt		0,5553	0,1047
49	kan	YHR059W	0,3043	0,0632
	nat	YNR052C	0,3909	0,1156
	kan nat		0,3396	0,0795
	wt		0,5414	0,0881
50	kan	YBL079w	0,2929	0,0912
	nat	YPR043W	0,4148	0,0731
	kan nat		0,2425	0,0705
	wt		0,5483	0,1083
51	kan	YLR373C	0,4568	0,0811
	nat	YBR126C	0,4830	0,0873
	kan nat		0,4675	0,0890
	wt		0,5506	0,0902
52	kan	YLR239C	0,5119	0,0883
	nat	YBR127C	0,3737	0,0917
	kan nat		0,3423	0,0860
	wt		0,5627	0,0910
53	kan	YNL139C	0,5575	0,0948
	nat	YDR529C	0,4975	0,0828
	kan nat		0,4837	0,0843
	wt		0,5758	0,1066
54	kan	YLR268W	0,4908	0,0840
	nat	YGL105W	0,3711	0,0693
	kan nat		0,3263	0,0886
	wt		0,5665	0,1002
55	kan	YDL115C	0,3325	0,1230
	nat	YGL167C	0,2986	0,0785
	kan nat		0,3218	0,0923
	wt		0,5245	0,0945
56	kan	YPL178W	0,4664	0,0856
	nat	YGL168W	0,4407	0,0809
	kan nat		0,3953	0,0977
	wt		0,5853	0,0880
57	kan	YGR081C	0,4512	0,0948
	nat	YER141W	0,4583	0,1118
	kan nat		0,3791	0,1073
	wt		0,5263	0,0935
58	kan	YLR318W	0,5269	0,1044
	nat	YER153C	0,4990	0,1003
	kan nat		0,4559	0,0787
	wt		0,5612	0,0870
59	kan	YGR188C	0,4318	0,0765
	nat	YLL033W	0,4114	0,0770
	kan nat		0,4420	0,0845
	wt		0,5594	0,0830
60	kan	YMR205C	0,3456	0,0833
	nat	YLR061W	0,3833	0,1013
	kan nat		0,3329	0,0771

	wt		0,5734	0,0832
61	kan	YNL107W	0,3960	0,0835
	nat	YLR067C	0,3787	0,0925
	kan nat		0,3655	0,1276
	wt		0,5677	0,0925
62	kan	YBR073w	0,4178	0,0849
	nat	YLR068W	0,3537	0,1239
	kan nat		0,4035	0,0727
	wt		0,5639	0,0894
63	kan	YIL009C-A	0,3150	0,1212
	nat	YLR087C	0,4680	0,0902
	kan nat		0,2611	0,1125
	wt		0,5399	0,1114
64	kan	YKL204W	0,4504	0,0909
	nat	YML007W	0,4399	0,0878
	kan nat		0,3330	0,0801
	wt		0,5443	0,0945
65	kan	YOR014W	0,3571	0,0939
	nat	YOL041C	0,4660	0,1062
	kan nat		0,3478	0,1131
	wt		0,5370	0,1106
66	kan	YBR173C	0,4809	0,0883
	nat	YEL051W	0,3632	0,0820
	kan nat		0,3215	0,0773
	wt		0,5291	0,0921
67	kan	YOR309C	0,5389	0,1075
	nat	YER068W	0,1942	0,0983
	kan nat		0,1911	0,1041
	wt		0,5461	0,1172
68	kan	YGR063C	0,2983	0,1121
	nat	YGR215W	0,3741	0,0915
	kan nat		0,2557	0,1074
	wt		0,5570	0,0982
69	kan	YIR009W	0,5113	0,0853
	nat	YHR060w	0,3969	0,0993
	kan nat		0,4013	0,0873
	wt		0,5324	0,0866
70	kan	YDR418W	0,5067	0,0800
	nat	YHR081W	0,3818	0,0714
	kan nat		0,3976	0,0795
	wt		0,5403	0,0865
71	kan	YOR182C	0,3052	0,0833
	nat	YLR139C	0,4824	0,0913
	kan nat		0,2466	0,0802
	wt		0,5455	0,1002
72	kan	YNL008C	0,5288	0,0904
	nat	YKL119C	0,2096	0,1417
	kan nat		0,1887	0,1254
	wt		0,5665	0,0848

73	kan	YHR183W	0,4153	0,1013
	nat	YDR159W	0,3568	0,0790
	kan nat		0,3696	0,0958
	wt		0,5429	0,0872
74	kan	YGL219C	0,4545	0,0830
	nat	YDR204W	0,4812	0,0790
	kan nat		0,3642	0,0745
	wt		0,5460	0,0925
75	kan	YMR304w	0,4575	0,1150
	nat	YPL086C	0,4445	0,0678
	kan nat		0,3250	0,0738
	wt		0,5561	0,0892
76	kan	YML010W-A	0,4438	0,0922
	nat	YGL071W	0,4731	0,0950
	kan nat		0,4143	0,0812
	wt		0,5466	0,0968
77	kan	YBR289W	0,4117	0,0780
	nat	YGL058W	0,4824	0,0764
	kan nat		0,3073	0,0947
	wt		0,4903	0,0895
78	kan	YMR089C	0,4938	0,0954
	nat	YGL064C	0,4997	0,0774
	kan nat		0,4367	0,0865
	wt		0,5404	0,0921
79	kan	YAR002W	0,4341	0,0766
	nat	YMR078C	0,4722	0,0878
	kan nat		0,3793	0,0797
	wt		0,5451	0,0889
80	kan	YER087W	0,3515	0,0742
	nat	YOL096C	0,4738	0,0799
	kan nat		0,2063	0,0835
	wt		0,5030	0,0851
81	kan	YJL140W	0,5211	0,0931
	nat	YHL025W	0,4013	0,0805
	kan nat		0,3917	0,0646
	wt		0,5406	0,1343
82	kan	YDR025W	0,5469	0,1096
	nat	YLR039C	0,4108	0,1079
	kan nat		0,4437	0,1037
	wt		0,5552	0,1110
83	kan	YPL036w	0,5498	0,0972
	nat	YML121w	0,5088	0,0986
	kan nat		0,4709	0,1006
	wt		0,5574	0,0998
84	kan	YOR241W	0,4282	0,0777
	nat	YPR036W	0,4520	0,0969
	kan nat		0,3841	0,0809
	wt		0,5059	0,1061
85	kan	YLR312W-A	0,4863	0,1143

	nat	YJR063W	0,4965	0,0940
	kan nat		0,4500	0,1006
	wt		0,5382	0,0978
86	kan	YBL099W	0,4364	0,0838
	nat	YCR071C	0,3284	0,0991
	kan nat		0,3246	0,0737
	wt		0,4873	0,0865
87	kan	YGR180C	0,3054	0,0975
	nat	YNL079C	0,4032	0,0814
	kan nat		0,3090	0,0655
	wt		0,4772	0,0919
88	kan	YMR272C	0,5284	0,0843
	nat	YKL139W	0,4083	0,0874
	kan nat		0,3108	0,0704
	wt		0,5413	0,0881
89	kan	YML032C	0,3599	0,0906
	nat	YDL033C	0,4961	0,0956
	kan nat		0,3894	0,1049
	wt		0,5504	0,0711
90	kan	YDL117W	0,4618	0,1091
	nat	YGL163C	0,4416	0,0769
	kan nat		0,3284	0,0916
	wt		0,5185	0,1010
91	kan	YKL170W	0,4644	0,1191
	nat	YLR069C	0,3772	0,0838
	kan nat		0,3172	0,0874
	wt		0,5383	0,0902
92	kan	YBR189W	0,4873	0,0811
	nat	YDR450W	0,3750	0,0855
	kan nat		0,4245	0,0971
	wt		0,5259	0,1122
93	kan	YNL001w	0,4423	0,0983
	nat	YJL102W	0,5133	0,0828
	kan nat		0,4763	0,0623
	wt		0,5366	0,1145
94	kan	YLR185W	0,3909	0,1242
	nat	YDR347W	0,3459	0,0983
	kan nat		0,3580	0,0660
	wt		0,5334	0,1090
95	kan	YJL189W	0,4810	0,1171
	nat	YPL239W	0,4308	0,0959
	kan nat		0,4044	0,0948
	wt		0,5479	0,0982
96	kan	YBR282W	0,5513	0,0933
	nat	YMR194W	0,4547	0,0790
	kan nat		0,4502	0,0979
	wt		0,5502	0,0838
